# Use of Indocyanine Green (ICG) to Assess Myocardial Perfusion and Territorial Distribution of Vein Grafts Implanted on Coronary Arteries in an *Ex-vivo* Porcine Model. A Potential Adjunct to Assist Revascularization Strategies and Training in Coronary Artery Bypass Grafting

**DOI:** 10.31083/RCM25778

**Published:** 2025-01-21

**Authors:** Cristiano Spadaccio, Antonio Nenna, Diletta Corrado, Carter Glenn, Antonio Panza, Russell Vester, Grzegorz Laskawski, David Rose, Louis Louis

**Affiliations:** ^1^Cardiac Surgery, University of Cincinnati Medical Center, Cincinnati, OH 45202, USA; ^2^Cardiac Surgery, Università Campus Bio-Medico di Roma, 00128 Rome, Italy; ^3^Cardiac Surgery, Blackpool Teaching Hospital - Lancashire Cardiac Center, FY3 8NP Blackpool, UK

**Keywords:** indocyanine green, fluorescent imaging, coronary artery bypass grafting, neoangiogenesis, arteriogenesis, graft patency, incomplete revascularization, fractional flow reserve

## Abstract

**Background:**

The fluorescent dye indocyanine green (ICG) has been used to identify anatomical structures intraoperatively in coronary artery bypass grafting (CABG). This study aimed to evaluate the feasibility of using ICG to assess graft patency and territorial distribution of myocardial reperfusion during CABG.

**Methods:**

Porcine arrested hearts (n = 18) were used to evaluate territorial distribution of native coronary arteries and of a coronary bypass constructed with porcine saphenous vein graft (SVG) using ICG. Coronary ostia were dissected and selectively cannulated for ICG injection. Sequential fluorescence was assessed in the epicardial coronary arteries, myocardium and coronary veins using an infrared-sensitive charge-coupled device (CCD) camera system. In a separate set of experiments, SVG was used for anastomosis in end-to-side fashion to a terminal obtuse marginal (OM) branch. This approach was used to avoid bias in the assessment of territorial distribution. The anastomosis was injected with ICG; graft patency and territorial distribution was assessed using an infrared-sensitive CCD camera system from 30 cm above the field, as previously described. Native circulation and SVG grafts were assessed using real-time video recording and fluorescence intensity mapping that was averaged into a graded scoring system. The heart was divided into functional regions: anterior wall, lateral wall, inferior wall and right ventricle. All experiments were performed in triplicates.

**Results:**

After ICG injection into the individual coronary ostia, perfusion of the native coronary artery was visible. Portions of the vessels embedded into the epicardial fat could be easily visualized on the surface of the heart and the dissection facilitated via fluorescence guidance. The territorial distribution reflected the expected regional perfusion. The SVG graft was anastomosed to an OM branch. ICG visualization allowed for assessment of graft patency excluding potential technical anastomosis problems or graft twisting or dissection. The myocardial perfusion observed in real-time confirmed regional distribution to the entire lateral wall and minimally to the inferior wall. These findings were confirmed in all the specimens used in the study.

**Conclusions:**

Besides assisting the identification of intramyocardial vessels, ICG can provide information on the native coronary circulation status and the territorial distribution of the perfusion before and after grafting. It enables visualization of collaterals and the territory of distribution subtended by a graft offering real-time assessment and guidance on the grafting strategy.

## 1. Introduction

Indocyanine green (ICG) is a water-soluble dye that has been used for its fluorescence properties as a contrast agent for imaging using near-infrared laser technology. It was approved for diagnostic use in humans in 1956 by the European Medicines Agency (EMA) and the Food and Drug Administration (FDA) for both interstitial and intravascular application [1]. It was initially used for lymphatic mapping to identify sentinel nodes in melanoma, allowing a selected lymphatic resection instead of an extensive resection. The use of ICG in general surgery is well established as it allows the visualization of bowel perfusion during resection and anastomosis, thus preventing anastomotic complications due to inadequate blood flow [2]. In vascular surgery, the main application is intra-operative guidance to visualize end-organ vascularization, as demonstrated by a study in peripheral arterial disease where fluorescent imaging with ICG was used as a tool to evaluate the perfusion of the foot before and after a revascularization procedure [3].

In cardiac surgery, fluorescent imaging with ICG has been utilized to improve visualization of intramural vessels or in patients with a considerable amount of epicardial fat to properly locate the course of coronary arteries. Another important application in heart surgery is the intraoperative assessment of graft patency, which is the prevalent determinant of freedom from reintervention and survival after coronary bypass surgery [4]. Very early graft failures had a reported incidence rate ranging between 4–12% in the modern series and are often correlated to technical problems at graft anastomosis sites, which are potentially correctable if recognized in a timely manner [5]. A randomized study, comparing intraoperative ICG graft assessment with transit-time flow measurement (TTFM), and validated with postoperative angiography, showed a better accuracy in the detection of clinically significant graft stenosis, with a sensitivity of 83.3%, a specificity of 100% and an added time to perform the test of 10 minutes [5]. ICG patency test of grafts in coronary artery bypass grafting (CABG) with systemic ICG injection has been used in the context of off pump beating heart surgery [4, 5, 6, 7]. Waseda *et al*. [8] analyzed the results on intraoperative fluorescent imaging of 137 patients and 507 grafts during off pump CABG. They studied the feasibility and accuracy of the use of ICG for graft assessment showing that full visualization was possible in 75% of the grafts due to technical and resolution reasons. Also, when compared to TTFM, 6 grafts considered acceptable were actually identified as failed when using the ICG test. Conversely, the rate of false negative (grafts acceptable during ICG testing thereafter found occluded at angiography) was 3.1%. Additionally, 4% of the grafts deemed acceptable with the ICG test showed poor flow at TTFM [8]. These results are in line with another study by Balacumaraswami *et al*. [9] demonstrating a 3.8% false negativity using the ICG assessment when compared to TTFM. Nevertheless, other groups confirmed in large series of more than 100 grafts studied, the clinical utility of this approach in identifying coronary anastomosis problems or graft failure. Reuthebuch *et al*. [6] in a series of 124 grafts, was able to identify graft failure intraoperatively in 3.7% of the cases, prompting graft revision. These findings have significant implications in terms of management and outcomes of CABG patients. In fact, the possibility of identifying graft failure related to technical issues allows for prompt revision during the operative phase, preventing significant ischemic events in the postoperative period, with tremendous impact on perioperative outcomes and patient prognosis. Additionally, differently from TTFM, ICG angiography allows a dynamic assessment of both anatomical and flow characteristics of the bypass, as shown by semiquantitive analysis performed on the washout timing of the ICG [8]. This might be at the root of the reported ability of ICG angiography to identify graft dysfunction related to flow competition or steal despite acceptable TTFM. The latter could have implications also on the long-term patency of the grafts and long-term clinical outcome, avoiding the need for repeat revascularization or a redo operation.

Additionally, many studies highlighted the safety of the use of ICG, as shown by lack of liver or metabolic derangement [6]. Lastly, ICG was suggested as a useful aid in identifying and locating the course of coronary arteries deeply situated in the epicardial fat or those with an intramural course [10].

Despite widely used in abdominal and vascular surgery to study end-organ perfusion [2, 3], little is known about the potential use of ICG fluorescence in understanding myocardial perfusion. In fact, all the studies previously discussed focus on the use of ICG angiography as a tool to study patency of the anastomosis but do not consider its ability to investigate territorial myocardial perfusion, as reported with abdominal and other organs. Perfusion studies with ICG have been performed in preclinical models of native coronary occlusion and demonstrated correlation between entity of flow restriction and myocardial perfusion defects [11, 12, 13]. However, these preclinical early studies were performed to understand the myocardial perfusion subtended by the native coronaries and aimed at studying in a quantitative fashion the effect of coronary occlusion. Therefore, they focused on the normal myocardial physiology and were centered on the perfusion analysis of the native circulation. Instead, the idea of using ICG to study the myocardial perfusion produced by a graft during a CABG revascularization procedure and of its topographical distribution in the different regions of the myocardium has been unexplored and might open up potential exciting avenues in coronary disease treatment and training.

The objective of this study was to assess the feasibility of intraoperative fluorescent imaging with ICG as a method to assess coronary and graft territorial distribution, beyond the simple coronary visualization and graft quality check.

## 2. Materials and Methods

### 2.1 Experimental Model

Porcine hearts (N = 18) were used in this preclinical CABG model to evaluate the potential of ICG to assess perfusion and territorial distribution of coronary arteries and vein grafts implanted on non-left anterior descending (LAD) targets.

To the first aim, coronary ostia of isolated porcine hearts were directly cannulated and injected with ICG (IC-Green®, Akorn Inc., Lake Forest, IL, USA) similarly to previously described methodology [11]. The ICG fluorescent dye was administered intravenously through a central venous line to ensure adequate mixing of the ICG. The dose of ICG was adapted to body weight (0.03 mg/kg body weight), injected as a slow bolus in a diluted saline solution of 10 mL. This allowed assessment of sequential fluorescence in the epicardial coronaries, myocardium, and washout via coronary veins. One heart was used to assess the epicardial coronary visualization. In the remaining hearts, selective occlusion of the proximal segments of the LAD, circumflex artery (CX) and right coronary artery (RCA) was achieved to observe differential distribution of perfusion. In brief, root aortic dissection was performed to expose proximal segment of the left main stem, LAD, CX and RCA. The latter were dissected from surrounding tissue and encircled in order to position an occlusive clamp. The coronary ostia were then selectively cannulated and injected as above described to assess territorial distribution of the single coronary. For simplicity the heart was regionally divided in functional regions: anterior wall, lateral wall, inferior wall and right ventricle.

To the second aim, porcine saphenous vein graft (SVG) were used to construct coronary anastomosis on a terminal obtuse marginal branch. We elected to avoid grafting LAD as the well known wide collateral network arising from this vessel might have biased the observation of the territorial distribution of the graft. Anastomosis wase performed as routine in end-to-side fashion using running 7–0 Prolene suture. The inverted SVG were directly injected with 10 mL of diluted ICG solution simulating the construction of a proximal anastomosis. Assessment of graft patency and territorial distribution was performed with Karl Storz-endoskope and the detection system as specified below (Tuttlingen, Germany). In this study the left internal thoracic artery was not utilized as it is unpractical to cannulate and directly injected in these *ex vivo* settings due to its small caliber. Additionally, using the left internal thoracic artery (LITA) as a free standing graft would not reflect the normal routine practice in CABG of utilizing this artery *in situ*, i.e., as a pedicle without detachment from its proximal origin in the subclavian artery.

Despite lacking the hemodynamic complexity and physiological conditions of a living body, this *ex vivo* model in arrested hearts was selected for this preliminary proof of concept study, permitting the assessment of ICG-related graft perfusion without the potential confounding biases related to native circulation competitive phenomena or rapid dye washout. In fact, the lack of native coronary disease in any porcine model could have affected the ability to study the actual perfusion of the implanted bypass. Nevertheless, the overarching aim of this research relies in the possibility of applying these findings in living conditions in the future. Further *in vivo* experiments on porcine models of coronary stenosis [14] and revascularization with bypass graft are currently being performed to validate and support the presently proposed findings.

The study was approved by local ethical committee for preclinical research.

### 2.2 Fluorescent Dye and Detection System

The technique is based on fluorescence properties of ICG (C_43_H_47_N_2_NaO_6_S_2_), which has maximal fluorescence and absorbance in the near infrared region. When illuminated with 806 nm light, ICG fluoresces and emits light at 830 nm.

The light necessary to excite the fluorescence is emitted by a laser with an output of 2.0 W at 806 nm, which illuminates the area where the porcine heart was located, positioned approximately 30 cm above. It is activated before the first pass of a bolus of ICG through the field of view. The laser is considered safe for both patient and operating staff and does not require eye protection, because the low energy laser (2.0 W) is dispersed in the ambient and remitted light and so has no potential health risks. In addition, there is not the risk of tissue warming due to the low power density of emitted light energy and the near-infrared light can penetrate soft tissue maximally by 1 to 2 mm.

**Fig. 1.**
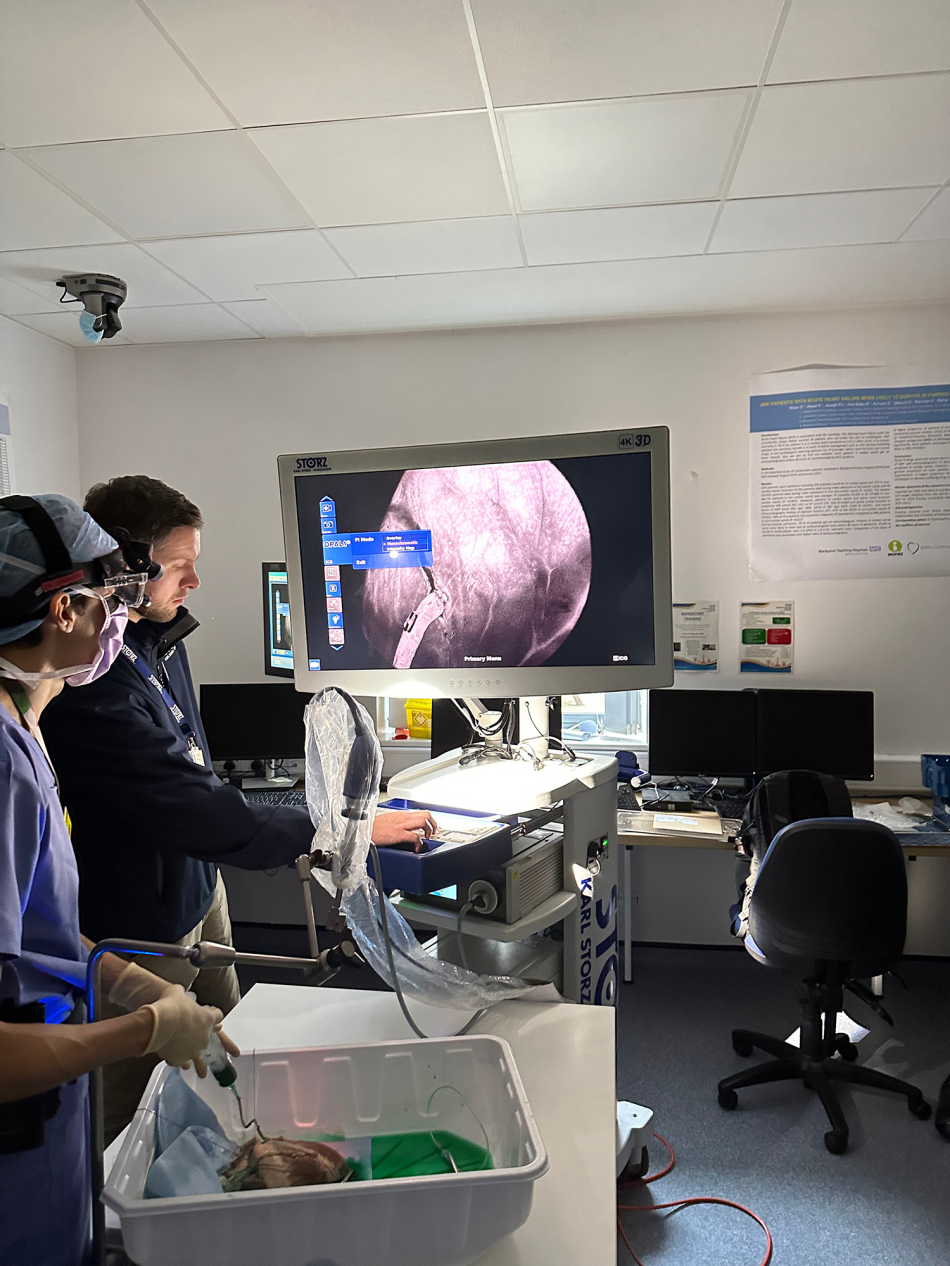
**Karl Storz Rubina® lens system and IMAGE1 S™ technology**. During the injection of ICG into the bypass graft, the light emitted by the laser device at 806 nm and 2.0 W excites the fluorophore and the fluorescent emitted light is captured by an infrared-sensitive charge-coupled device (CCD) camera system. The image is clearly displayed in a 4K monitor with the opportunity to obtain information about the intensity of the fluorescent signal (intensity map) and a pure modality of near infrared (monochromatic) to distinguish the contour of the anatomical structure. The possibility to register the fluorescent signal assures the quantification of parameters which are helpful for the stenosis measurement and the control of quality of graft-coronary anastomosis. ICG, indocyanine green. The persons approved the use of this photograph for publication.

The fluorescent light emitted by excited ICG is captured by an infrared-sensitive charge-coupled device (CCD) camera system shown in Fig. 1 (Rubina® lens system and IMAGE1 S™ technology, Karl Storz-endoskope, Tuttlingen, Germany). This camera is integrated with a band pass filter for the selective transmission of light at 830 nm, which is the maximum emission of ICG. The fluorescent cardiac images were displayed and recorded in real-time as the ICG passes through the field of view on a 4K monitor, with the possibility of seeing 2D and 3D images. In addition, this system offers information about the intensity of the fluorescent signal (intensity map) and a pure modality of near infrared (monochromatic) to clearly distinguish the delimitation and the contour of anatomical structures. Intensity maps allow for measurement of the intensity of the fluorescent signal in different regions of interest. The system reports the fluorescence signal as fluorescence units (F.U.).

For the assessment of the native epicardial coronary distribution, real-time video recording and fluorescence intensity map was obtained. SVG graft distribution was analyzed similarly. For simplicity the numeric measurements pertinent to the different regions of interest, have been averaged into a grading score system in order to provide a simple semiquantitive scale index (0 to 4; 0 = no signal; 1 = mild signal; 2 = moderate signal; 3 intense signal; 4 = very strong signal). Specifically, the range of F.U. from 0 to 1000 was assigned to category 0 = no signal: the F.U. range 1001–3000 assigned to category 1 = mild signal, range 3001–5000 assigned to category 2 = moderate signal, range 5001–7000 to category 3 = intense signal, range 7000–9000 to category 4 = very strong signal. The zero values have been indexed and normalized considering the baseline level of myocardial autofluorescence measured in resting conditions before the injection of the ICG. This type of semiquantitative approach has been previously described and validated in clinical studies assessing the patency of the bypass graft after ICG injection in humans [8]. The heart was divided into functional regions and a score was assigned according to the intensity of the perfusion provided by the system. All experiments were performed in triplicates. Results of both the absolute measured value and of the graded system are reported.

### 2.3 Statistical Analysis

All measurements were performed in triplicate on each heart and each region was mapped. Normality criteria were checked with graphical and numeric (Shapiro–Wilk test) tests. Continuous data are shown as median and [1st to 3rd quartile] and compared using non-parametric tests (Wilcoxon test for paired data). A *p* value < 0.05 was considered statistically significant. Statistical analysis was performed with STATA version 17 (StataCorp LLC, College Station, TX, USA).

## 3. Results

### 3.1 Native Epicardial Coronaries Imaging and Perfusion

**Fig. 2.**
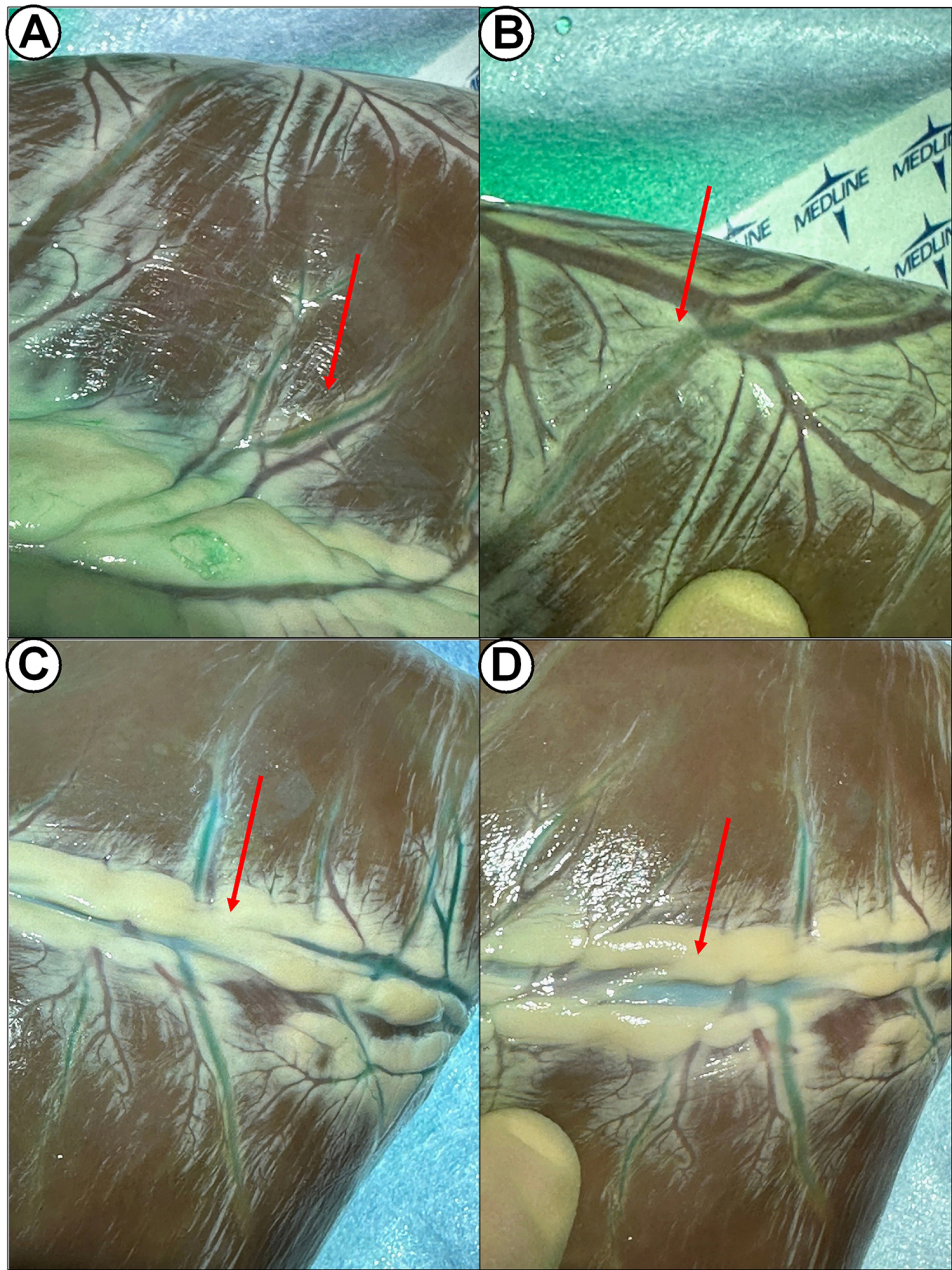
**Visualization of coronary arteries after ICG injection**. The injection of ICG into coronary ostia via direct cannulation shows coronary stenosis or occlusion and, when there is a thick layer of epicardial fat, the fluorescence of the dye may guide the dissection of fat tissue to show the coronary arteries (red arrows). Therefore, it is possible to visualize in real time the perfusion of intramural coronary arteries to facilitate dissection (A, B: lateral wall; C, D: inferior wall). This technique allows an initial assessment of the course and the occlusions of coronary arteries. ICG, indocyanine green.

**Fig. 3.**
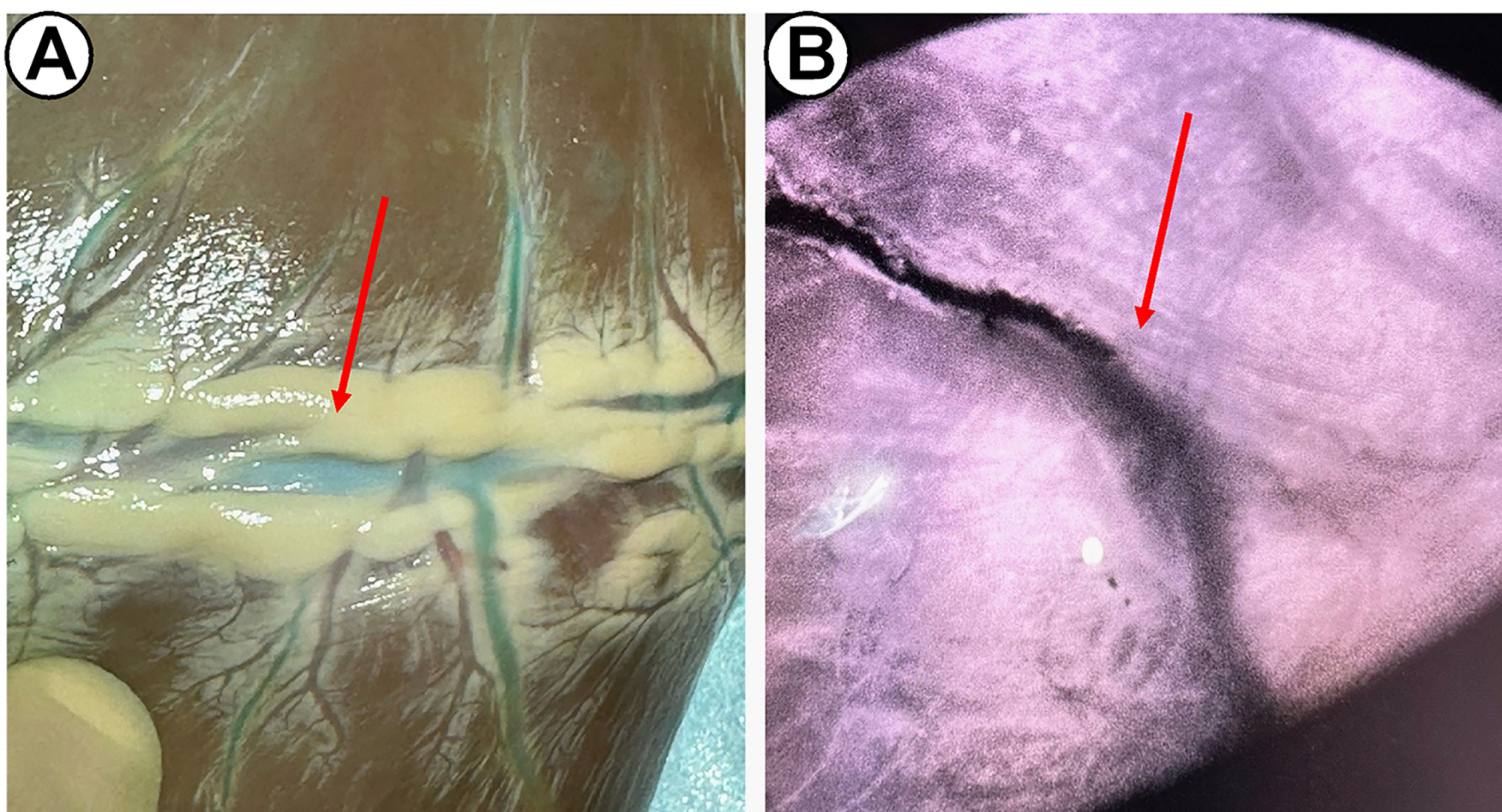
**Visualization of the native coronary arteries with ICG injection**. Anatomic visualization (A) and fluoroscopic signal (B). Red arrow: native coronary artery. ICG, indocyanine green.

Fig. 2 and Video 1 show the real time perfusion of native coronary arteries after direct injection into the coronary ostia. Portions of the vessels embedded into the epicardial fat could be easily visualized on the surface of the heart and the dissection facilitated via the fluorescence guidance (Video 1) (Fig. 3).

**Video 1.** **Real time perfusion of native coronary arteries after direct injection of ICG into the coronary ostia**. ICG, indocyanine green. The embedded movie may also be viewed at https://doi.org/10.31083/RCM25778.

Table 1 shows the perfusion scoring obtained by selectively injecting the isolated native LAD, CX or RCA. The territorial distribution reflected the expected regional perfusion subtended by the different arteries. Namely, LAD extensively perfused the anterior, septal and partially the lateral wall, where the CX injection produced myocardial enhancement of the lateral and inferior regions. Lastly, the RCA perfused the right territories and the inferior wall.

**Table 1.** **Perfusion scoring and territorial distribution of native coronary arteries and graft**.

	Anterior wall	Lateral wall	Inferior wall	Right ventricle
LAD	4	2	1	0
CX	0	4	1	0
RCA	0	0	2	4
Graft (SVG on OM)	0	4	1	0

Perfusion is semi-quantitatively graded 0 (absence) to 4 (maximal) after ICG administration. Grades are based on selective injection of native coronary artery or selective graft injection. LAD, left anterior descending artery; CX, circumflex artery; RCA, right coronary artery; SVG, saphenous vein graft; OM, obtuse marginal artery.

### 3.2 Venous Graft Bypass Imaging and Perfusion

Considering the potential territorial overlap and collateralization of the LAD, the obtuse marginal 1 (OM1) branch was identified and grafted as it was considered to provide a more terminal irroration with a potentially less spurious signal in terms of regional perfusion.

**Fig. 4.**
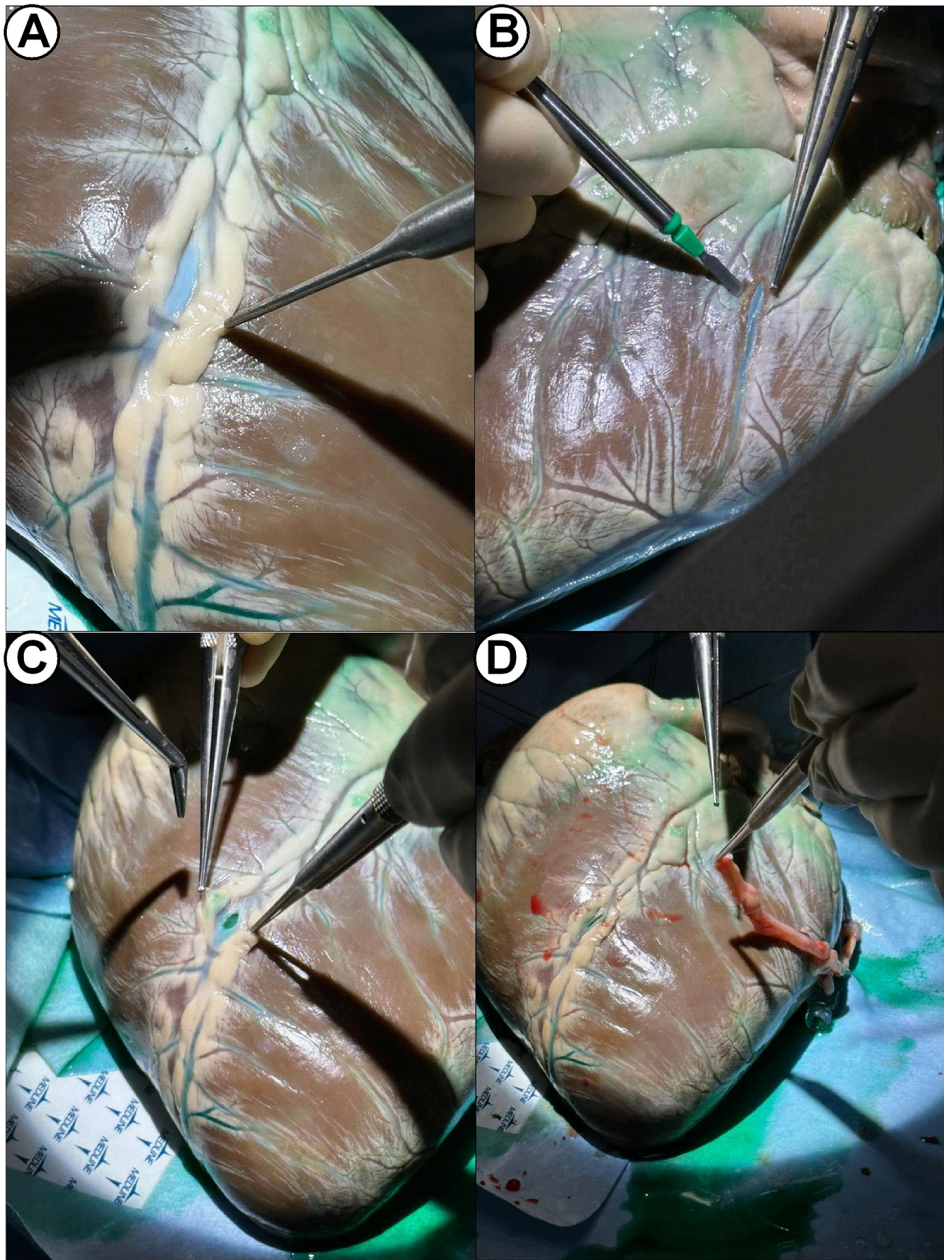
**Identification of target coronary artery, removal of epicardial tissue and end-to-side anastomosis with the aid of ICG injection**. Vessel identification (A), opening (B,C) and anastomosis (D). ICG, indocyanine green.

**Fig. 5.**
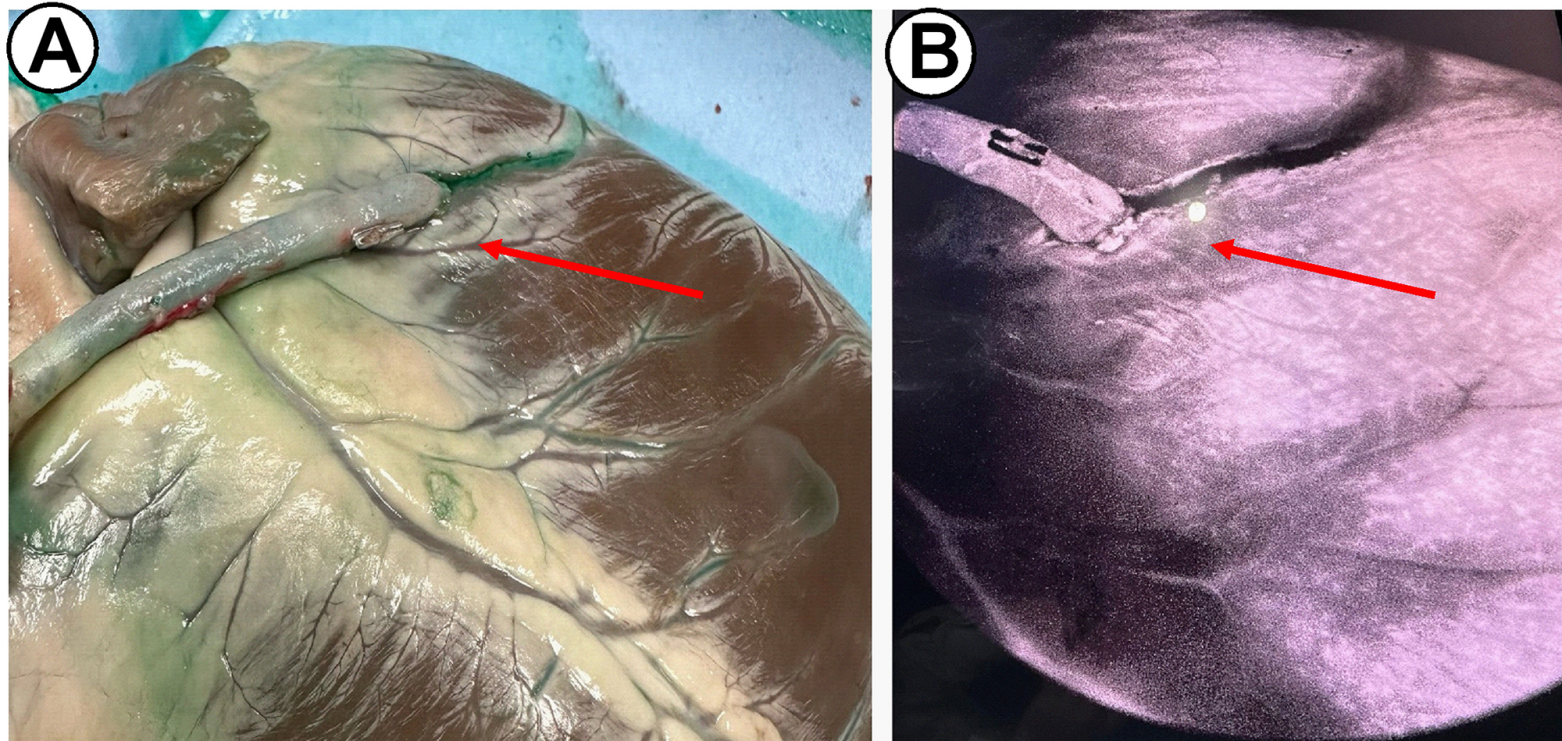
**Visualization of the saphenous graft to obtuse marginal perfusion with ICG injection**. Anatomic visualization (A) and fluoroscopic signal (B). Red arrow: graft anastomosis. ICG, indocyanine green.

**Fig. 6.**
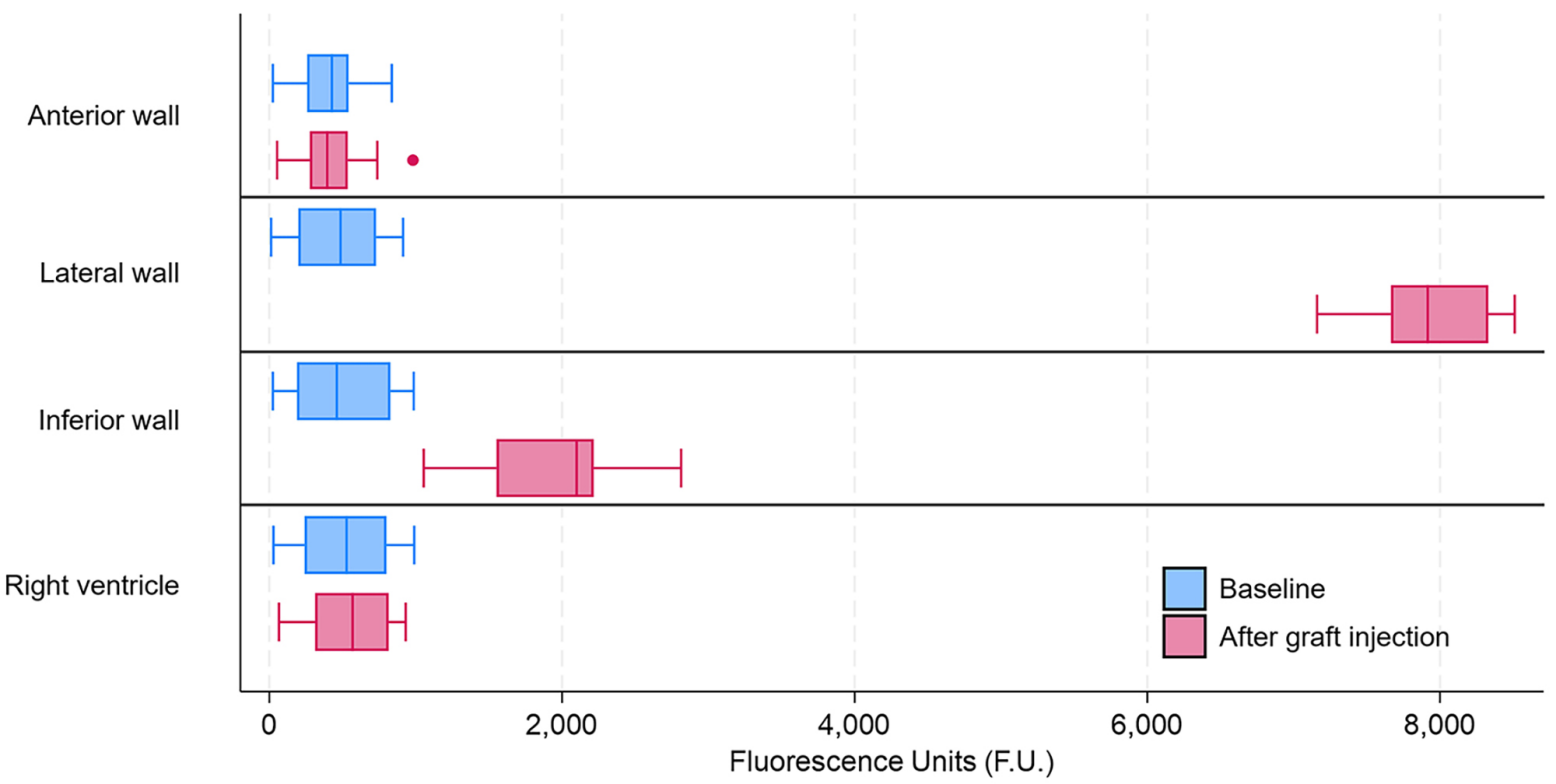
**Fluorescence and territorial distribution at baseline and after graft injection**. Box-and-Whisker plot is formatted according to Tukey.

As shown in Fig. 4 and Video 2, immediate patency of the graft could be confirmed excluding potential technical anastomosis problems or graft twisting or dissection (Video 3). The myocardial perfusion observed in real-time confirmed regional distribution to the entirety of the lateral wall (4+) and minimally to the inferior wall (1+) (Table 1) (Fig. 5). Values of fluorescence signal at baseline and after injection are reported in Table 2 and Fig. 6. These findings were confirmed in all the specimens used in the study.

**Video 2.** **Evaluation of immediate graft patency and visualization of regional distribution of myocardial perfusion**. The embedded movie may also be viewed at https://doi.org/10.31083/RCM25778.

**Video 3.** **Evaluation of immediate graft patency, anastomosis check and backflow to the aortic root**. The embedded movie may also be viewed at https://doi.org/10.31083/RCM25778.

**Table 2.** **Fluorescence units and territorial distribution at baseline and after graft injection**.

N = 18	Baseline	After graft injection	*p* value (paired test)
Anterior wall	427 (258–541)	396 (277–536)	0.744
Lateral wall	486 (198–729)	7916 (7665–8330)	0.001
Inferior wall	461 (189–827)	2100 (1553–2216)	0.001
Right ventricle	528 (241–800)	569 (313–815)	0.161

Data are shown as median and [1st to 3rd quartile].

## 4. Discussion

In this preliminary study, we demonstrated the feasibility of using ICG to study not only patency and anastomosis quality but also the territorial distribution of vein grafts.

In fact, while confirming previous findings of the use of ICG to facilitate epicardial coronary visualization and intraoperatively assess graft quality, this is the first preclinical report demonstrating the potential use of ICG to assess the territorial perfusion obtained via selectively grafting native coronaries. With this technique it is possible to observe the area of distribution of the native circulation and the one subtended by an implanted vein graft. ICG allows visualization of the surface of the myocardium vascularized by the coronary artery anastomosed to the graft.

Despite preliminary and needing confirmation in additional *in vivo* studies, these findings have several potential implications.

Firstly, this approach can significantly facilitate the identification of targets in the cases of coronary arteries which have taken an intramural course. This can be particularly relevant in the field of minimally invasive or totally endoscopic or robotic coronary artery bypass surgery. Secondly, the immediately intraoperative quality assessment provided by this approach might potentially mitigate the risk of early graft failure due to technical error by allowing early recognition of anastomotic problems and prompt correction or revision. Additionally, the accuracy of patency and flow evaluation might be even higher than the routinely used TTFM, as already shown by a randomized clinical trial and other retrospective studies demonstrating 100% specificity and a false negative rate of less than 5% when compared to TTFM [5, 6, 7, 8].

Most importantly, beside the quality control of the anastomosis, this preliminary study potentially introduces a novel concept relative to the intraoperative assessment of the regional myocardial perfusion obtained with each of the bypass grafts performed. Native territorial distribution could be immediately visually observed before and after bypass graft construction and potentially even quantified by means of background-subtracted peak fluorescence intensity (BSFI) and slope of fluorescent intensity (SFI), as shown in preclinical models of native coronary occlusion [11, 12, 13].

The possibility of an immediate real-time *in situ* assessment of the native circulation and of the effect of coronary revascularization might provide invaluable information not only on the basic mechanisms of coronary perfusion and on the impact of surgical revascularization on the native coronary circulation, but also could be crucial to guide the revascularization strategy itself.

In fact, it is widely known that native coronary collateral circulation is a dynamic vascular network with high remodeling potentials which can act as a natural bypass mechanism [15]. Also, the collateral network in patients with coronary artery disease (CAD) provides more than half of normal myocardial perfusion [16] and the presence of coronary collateralization is a long-term survival predictor in patients with CAD [17]. The interference of surgical revascularization with the dynamic equilibrium of native collateral circulation is poorly understood. However, studies have shown that CABG can induce degeneration of native collaterals [14, 18]. On the other side, CABG is thought to induce local neoangiogenesis via the release of paracrine mediators from the arterial or venous conduits used. In fact, graft-derived perfusion has been supposed to result in the progressive spreading of neo-angiogenesis from the grafted territory towards other territories overlapping and assisting the process of revascularization of ischemic territories [18, 19, 20]. The *in situ* visual observation of the territorial coverage of a coronary bypass graft could assist in understanding these mechanisms but could also provide visual feedback of the regional perfusion achieved before and after each bypass. This could in turn potentially allow tailoring of the revascularization strategy and the number of grafts to be performed in order to achieve a complete revascularization. In fact, when translated into the routine surgical practice ICG-based visualization of the regional perfusion achieved by each graft could assist the surgeon and provide real-time guidance on the revascularization strategy, especially when grafting multiple borderline lesions in target vessels in proximity and serving similar areas of the heart, or in cases of well-collateralized areas. For example, the surgeon could first assess the native coronary circulation status (*in situ* angiography), then once the first graft is performed, the ICG-perfusion could guide the following grafting strategy, showing in real time the area served by the graft just constructed. If a bypass graft is able to serve a large territory overlapping with the one pertaining to another stenotic vessel close by, the surgeon could safely elect not to perform an additional bypass as the previously constructed graft is already providing enough perfusion. This would reduce the length of the operation and the risk of the surgery without compromising the completeness of the revascularization, with significant potential impact on clinical outcomes. Conversely, in the case of borderline lesions, ICG could reveal lack of adequate perfusion and could support the decision-making of angiographically bypassing non-significant stenotic vessels.

These concepts intertwine with those of the emerging field of “functional” myocardial revascularization as opposed (or as complement) to “anatomical” revascularization in decision making for CABG [21, 22, 23, 24, 25]. The visual assessment of the *in situ* native territorial distribution (i.e., native circulation and collateral) and the one gained with each bypass graft could complement the preoperative information on the functional significance of a coronary stenosis provided by fractional flow reserve (FFR) measurements. The overarching aim of this preliminary study was indeed to explore ICG as a tool to achieve a better understanding of the “functional and topographical” perfusion effects of a coronary bypass graft. This can be instrumental to inform the revascularization strategy when combining with data from FFR, angiography, and myocardium viability. Another intriguing field and avenue regarding chronically occluded vessels, especially in territories where collateralization cannot be adequately visualized using angiography. ICG myocardial perfusion could assist in the decision of grafting totally occluded vessels and in assessing the actual effect of a bypass graft in these region.

Clearly, this is a preliminary study and, despite being promising, these results should be confirmed in further preclinical and clinical settings, especially introducing the elements relative to hemodynamics and native circulation characteristics. Therefore, at present the majority of the above considerations remain speculative. However, the possibility of rapid clinical application is not far-reaching given the safety profile of ICG [26] and its approved use clinical settings. Importantly, ICG immediately binds to plasma proteins and remains intravascular after systemic injection without risk of extravasation due to the non-covalently (95%) bound albumin, its major protein carrier. It has a half-time of 3–5 minutes (mean half-time of 2.4 minutes) and it is rapidly eliminated by the liver in 15–20 minutes, a characteristic that allows repeated injections of ICG. It is then secreted into the bile without chemical changes. Therefore, the lack of renal excretion renders it a safe fluorophore to use in cardiac surgery due to the great incidence of renal function impairment and acute or chronic kidney failure in patients affected by coronary atherosclerotic disease. Clinical experience with ICG-based evaluation of graft patency has been already documented by Reuthebuch *et al*. [6] and Taggart *et al*. [7].

Moving forward, *in vivo* preclinical and clinical studies are required to validate these findings. The *in vivo* dynamic conditions would present further challenges related to blood flow, dye washout, hemodynamics, competitive flow with the native circulation, visualization of the perfused region. Of note Waseda *et al*. [8] and Reuthebuch *et al*. [6] in their study on graft patency assessment have highlighted the possibility of not fully visualizing the blood vessels injected with ICG due to anatomical issues and limitations of the detection system. The use of dedicated tissue perfusion scanning systems, such as the SPY Elite intraoperative fluorescence imaging system (Stryker, MI, USA) could provide improved regional mapping of the perfused area and optimize the methodology and the information gainable during surgery. If confirmed by *in vivo* studies, this technology might hold the promise to represent an important adjunct in coronary revascularization surgery. Importantly, as previously discussed this ICG-based approach could assist grafting strategy, i.e., number of grafts to be performed to achieve complete revascularization, with potential significant impact on clinical outcomes. Future clinical studies should indeed not only focus on the immediate postoperative outcomes (acute graft failure, rate of coronary anastomosis revision, postoperative myocardial infarction), but also assess long-term postoperative clinical outcomes including freedom from symptoms, freedom from repeat revascularization, freedom from major cardiovascular events, and survival. These outcomes should be compared to cases in which ICG technology is not used. Additionally, an assessment of revascularization completeness would be important to validate the ability of this method to guide grafting strategy achieving optimal myocardium reperfusion.

Nevertheless, for its intrinsic ease of use and visual information provided, the use of ICG can be instrumental for training purposes, as it can assist in teaching coronary artery territory distribution, refining the ability to assess anastomosis functionality and recognize technical errors, but also understanding the importance of grafting strategy according to the entity of native coronary stenosis and preventing competitive flow.

### Limitations

This was an exploratory preliminary study, and authors acknowledge several limitations. The main limitation of this study is the application on an *ex-vivo* preclinical model only, constituted by arrested porcine hearts. Considering the nature of the model, it is possible that the territorial perfusion observed could be due to extravasation of the dye as the microvascular permeability regulation could have been lost in a not alive organ. Unfortunately, it was not possible to visualize the dye at fluorescence microscopy in the tissue to determine whether the dye is intravascular only or in the extravascular tissues due to the short half-life of ICG. In fact, the fluorescence would decay rapidly making difficult to complete a timely assessment on fresh frozen section, and the routine paraffine inclusion for regular histology would bias the visualization due to tissue autofluorescence. *In vivo* larger experiments may obviate to this problem. However, it is important to consider that the *in-vivo* application of ICG may modify the optical fluorescence visualization parameters due to changes in blood pressure, cardiac output and heart rate. In addition, the movements of a beating heart may modify the absolute values of the intensity of fluorescence, even if the visualization of coronary arteries remains clear, as demonstrated by the application of this intraoperative technique in off-pump CABG. This *ex vivo* model in arrested hearts was selected for this preliminary proof of concept study as permitting the assessment of ICG-related graft perfusion without the potential confounding biases related to native circulation competitive phenomena or rapid dye washout. In fact, the lack of native coronary disease in any porcine model could have affected the ability to study the actual perfusion of the implanted bypass. However, with the view of translating these findings in the clinical practice, further *in vivo* experiments are warranted to confirm the findings. These experiments should take into consideration the mentioned elements related to an “*in vivo* condition” including hemodynamics, blood flow characteristics, dye washout, heart rate, presence of competitive flow, impact of coronary stenosis. With this in mind, a close simulation of the clinical reality of CABG with a porcine model of coronary stenosis with ameroid constrictors [27] and further revascularization is being used to validate and support the presently proposed findings.

Despite the main aim of this study being to study myocardial perfusion, rather than graft patency, it would have still been desirable to provide comparative data with other methods of graft patency assessment, such as TTFM. However, considering the model was on arrested heart with hand-held injection of ICG, it would have been unreliable to measure the flow on the implanted bypass as this would not have been sustained by physiological circulation. The sample size is relatively low but considering the preliminary nature of the study and the low-grade variability in porcine anatomy, the triplicate repetitions used in the experimental settings, should have accommodate for the potential bias.

## 5. Conclusions

In this study we demonstrated alongside the possibility of improving visualization of the coronaries, the potential use of ICG to show the native coronary circulation status and the territorial distribution of the perfusion induced by a graft. This tool holds promise for further exciting avenues for “*in-situ*” territorial myocardial perfusion assessment before and after a bypass graft. Ideally, this method could provide real-time guidance on the revascularization strategy when grafting multiple borderline lesions in target vessels in close proximity and serving similar areas of the heart, or in cases of well-collateralized areas [28, 29, 30]. Indeed, the surgeon could first assess the native coronary circulation status (*in situ* angiography), then once the first graft was performed, the ICG-perfusion could guide the following grafting strategy, showing in real time the area served by the graft just constructed.

Additionally, such an ICG-based approach could help to unravel the intricate conundrum of the functional significance of a coronary lesion and the consequences of revascularizing heart territories on an FFR base or guidance.

Lastly, it would shed light on the immediate effect in terms of perfusion after grafting, providing further explanations to the described phenomena of flow competition or degeneration of native collateralization after CABG.

Future *in vivo* studies are required to confirm these findings and further elaborate on the potential use of ICG in CABG settings.

## Data Availability

All data reported in this paper will also be shared by the lead contact upon request.

## Abbreviations

BSFI, background-subtracted peak fluorescence intensity; CABG, coronary artery bypass grafting; CAD, coronary artery disease; CX, circumflex artery; FFR, fractional flow reserve; FI-ICG, fluorescence imaging - indocyanine green; LAD, left anterior descending artery; LITA, left internal thoracic artery; ITA, internal thoracic artery; NO, nitric oxide; RCA, right coronary artery; SFI, slope of fluorescent intensity; SVG, saphenous vein graft.
